# A space for lattice representation and clustering

**DOI:** 10.1107/S2053273319002729

**Published:** 2019-04-30

**Authors:** Lawrence C. Andrews, Herbert J. Bernstein, Nicholas K. Sauter

**Affiliations:** a Ronin Institute, 9515 NE 137th Street, Kirkland, WA 98034-1820, USA; b Rochester Institute of Technology, c/o NSLS-II, Brookhaven National Laboratory, Upton, NY 11973-5000, USA; c Ronin Institute, c/o NSLS-II, Brookhaven National Laboratory, Upton, NY 11973-5000, USA; d Lawrence Berkeley National Laboratory, 1 Cyclotron Road, Berkeley, CA 94720, USA

**Keywords:** unit-cell reduction, Delaunay, Delone, Niggli, Selling, clustering

## Abstract

Algorithms for quantifying the differences between two lattices are used for Bravais lattice determination, database lookup for unit cells to select candidates for molecular replacement, and recently for clustering to group together images from serial crystallography. It is particularly desirable for the differences between lattices to be computed as a perturbation-stable metric, *i.e.* as distances that satisfy the triangle inequality, so that standard tree-based nearest-neighbor algorithms can be used, and for which small changes in the lattices involved produce small changes in the distances computed. A perturbation-stable metric space related to the reduction algorithm of Selling and to the Bravais lattice determination methods of Delone is described. Two ways of representing the space, as six-dimensional real vectors or equivalently as three-dimensional complex vectors, are presented and applications of these metrics are discussed. (Note: in his later publications, Boris Delaunay used the Russian version of his surname, Delone.)

## Abstract 2

Algorithms for defining the difference between two lattices are described. They are based on the work of Selling and Delone (Delaunay).

## 1. Introduction   

Andrews *et al.* (2019 ▸) discuss the simplification resulting from using Selling reduction as opposed to using Niggli reduction. Here we continue that discussion with information on the space of unit cells and the subspace of reduced cells as the six-dimensional space 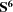 of Selling inner products.

Algorithms for quantifying the differences among lattices are used for Bravais lattice determination, database lookup for unit cells to select candidates for molecular replacement, and recently for clustering to group together images from serial crystallography. For crystallography, there are many alternative representations to choose from as a basis for distance calculations. Andrews *et al.* (1980 ▸) discussed 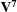, a perturbation-stable space in which, using real- and reciprocal-space Niggli reduction, a lattice is represented by three cell edge lengths, three reciprocal cell edge lengths and the cell volume, which was proposed for cell database searches, but which has difficulties when used for lattice determination. Andrews & Bernstein (1988 ▸) discussed 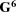 that uses a modified metric tensor and a search through 25 alternative reduction boundary transforms (Gruber, 1973 ▸) to work in a satisfactory manner both for database searches and for lattice identification in the presence of experimental error. Andrews & Bernstein (2014 ▸) discussed sewing together regions of the fundamental region of 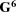 under Niggli reduction at 15 boundaries. Andrews *et al.* (2019 ▸) presented the simplest and fastest currently known representation of lattices as the six Selling scalars obtained from the dot products of the unit-cell axes in addition to the negative of their sum (a body diagonal). Labeling these 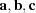 and 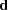 (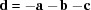), the scalars are
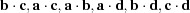
(where, *e.g.*, 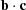 represents the dot product of the 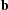 and 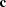 axes). For the purpose of organizing these six quantities as a vector space in which one can compute simple Euclidean distances, we describe the set of scalars as a vector, 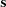, with components, 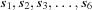. The cell is Selling reduced if all six components are negative or zero (Delone, 1933 ▸). Minimizing among distances computed from alternate paths between Selling-reduced cells with appropriate sewing at the six boundaries of the Selling-reduced fundamental region of 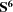 yields a computationally sound metric space within which to do lattice identification, cell database searching and serial crystallography clustering.

We define two equivalent spaces related to the Selling reduction: the space of six-dimensional real vectors, 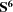, or equivalently the space of three-dimensional complex vectors 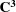:
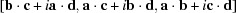
or
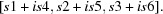
Although 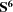 and 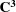 simply reorganize the same data, some operations are simpler to visualize in one space than the other. In some cases, we will choose to show only the simpler one.

The objective of this paper is to explain how to compute the distances between lattices using 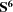 and 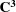.

## The space 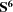   

2.

For a Bravais tetrahedron (Bravais, 1850 ▸) with defining vectors 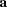, 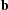, 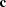, 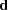 (the edge vectors of the unit cell plus the negative sum of them), a point in 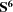 is
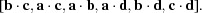
A simple example is the orthorhombic unit cell (10, 12, 20, 90, 90, 90) (a, b, c, α, β, γ). The corresponding 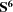 vector is 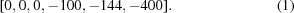
The scalars in 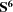 are of a single type, unlike cell parameters (lengths and angles) and unlike 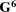 (squared lengths and dot products). Delone *et al.* (1975 ▸) state ‘*The Selling parameters are geometrically fully homogeneous*’.

Because there is no crystallographic reason to favor one ordering of 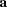, 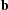, 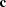, 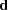 over another, for any given Selling-reduced cell there are 24 fully equivalent presentations as 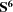 vectors generated by the 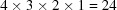 possible permutations of 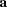, 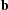, 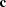, 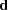 (Andrews *et al.*, 2019 ▸). To compute a distance between two different Selling-reduced cells, the least we will need to do is to compute the minimum of the distances between one of the cells and the 24 possible permutations of the other (Andrews *et al.*, 2019 ▸).

In addition, because Selling-reduced cells are defined as having only zero or negative scalars, the space has boundaries at the transitions to positive scalars. Therefore, if either of the two different Selling-reduced cells is in the vicinity of a boundary, we also need to consider the path changes that may arise from the reduction steps at that boundary. Additional, lower-dimension boundaries may be implied when scalars have equal values, but explicit consideration of those in addition to the permutations and sign-transition boundary transformations does not appear to be needed.

Some of the properties of 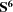 are simple. The six base axes are orthogonal, unlike those of 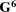 (Andrews & Bernstein, 2014 ▸). For example, the matrix projecting onto the 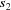 axis is
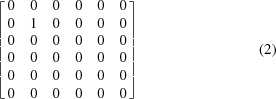
and the matrix projecting onto the five-dimensional polytope (the ‘perp’) spanned by 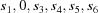 orthogonal to the *s*_2_ axis at *s*_2_ = 0 is 
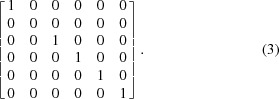



### The reflections in 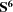   

2.1.

The 24 equivalent positions (Andrews *et al.*, 2019 ▸) in 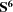 have corresponding matrices designed to act on 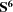 vectors to map them into crystallographically equivalent vectors. For convenience, they are all listed in Table 1 ▸. The structure of the set is clearer in 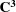. See Table 2 ▸, which presents the reflections in the same order.

The unsorted nature of Selling reduction implies that distance calculations will need to consider the reflections. Even if a usable sorting of points in the fundamental unit were created, at least some of the reflections would still be required for near-boundary cases.

### Reduction in 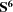   

2.2.

Lattice reduction is quite simple in 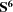 (Andrews *et al.*, 2019 ▸), but it has a clearer structure in 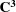, so it will be treated there (Section 3.2). Because of the simple nature of 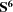, the inverse of each reduction operation is the same as the unreduction operation, so we term them edge transforms. The matrices in 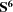 are unitary, so the metric is the same in each region. However, the transformation matrices are not diagonal, with the result that the boundaries are not simple mirrors.

We present the edge transforms as matrices, two for each scalar; the second line for each is the alternate choice of which pair to exchange [copied from Andrews *et al.* (2019 ▸)].

For the 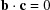 boundary 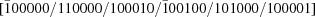
or 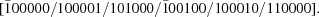



For the 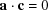 boundary 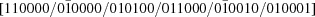
or
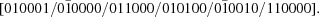



For the 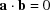 boundary 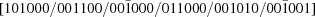
or
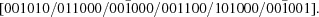



For the 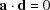 boundary 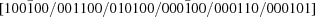
or 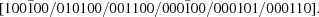



For the 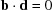 boundary 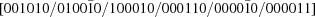
or 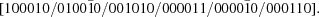



For the 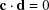 boundary 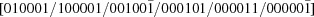
or 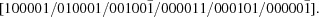



### The boundaries in 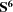   

2.3.

The first type of boundary in 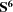 is the polytope where one of the six axes is zero. [Contrast this with 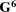 (Andrews & Bernstein, 2014 ▸), which has 15 boundaries of several types.] Obviously, the zeros correspond to unit-cell angles of 90°. In 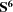, the zeros mark the regions where components change from negative to positive, *i.e.* the place where cells become non-Selling reduced. A second kind of boundary is where certain ‘opposite’ pairs of scalars are equal; this is more easily visualized in 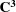 where those pairs are just the real and imaginary parts of one complex scalar. These are handled as ‘reflections’ (see Sections 2.1 and 3.1).

The consequence for distance calculations will be that the reduction operations will be involved in the distance computations.

## The space 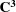   

3.

Alternatively, the space 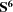 can be as represented as 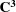, a space of three complex axes. 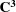 has advantages for understanding some of the properties of the space. When we compose 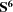 of the scalars 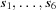, the components of 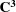 are the pairs of ‘opposite’ (Delone *et al.*, 1975 ▸) scalars. In terms of the elements of 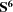, a unit cell in 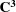 is 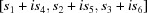 . The 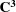 presentation of the vector (1) from Section 2 is 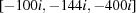.

### The reflections in 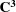   

3.1.

The 24 reflections of the scalars correspond to 24 reflection operations in 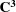. First, any pair of 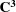 coordinates may be exchanged. The other reflection operation is the exchange of the real and imaginary parts of each member of any pair of 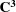 coordinates. We use 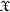 (for ‘eXchange’) to denote this operation. For example, 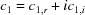 and 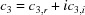 can transform to 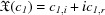 and 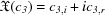. For complex numbers such an exchange can be effected by taking the complex conjugate and multiplying by *i*, so 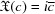.

Combining the exchange operation with the coordinate interchanges in all possible combinations gives the 24 reflections (including the identity).

Representing the operation of interchanging the real and imaginary parts of a complex number by 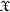, the 24 reflections in 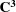 as permutations of 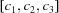 are given in Table 2 ▸.

### Reduction in 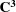   

3.2.

In 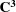, reduction has a more ordered form than in 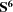. Consider a general point in 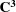 with components 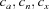. For descriptive purposes, let us assume that the imaginary part of 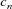 is the sole positive scalar, the one we must reduce.

Step 1: subtract the imaginary part of *c_n_* from the real part and change the imaginary part of *c_n_* to its negative value.

Step 2: add the original value of the imaginary part of *c_n_* to the real and the imaginary parts of *c_a_* and *c_x_*.

Step 3: exchange the real part of *c_a_* with the imaginary part of *c_x_*. (The alternative choice of exchanging the real part of *c_a_* with the imaginary part of *c_x_* is also valid.)

The reduction operations do not commute, which will add complexity to distance calculations (see Section 4 below). The two choices are related by one of the reflection operations. For distance calculations, all of the reflections must be considered, so the choice will not matter in the end.

### An asymmetric unit in 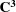   

3.3.

The fundamental unit in 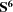 and 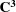 is chosen to be the region where all six scalars are zero or negative. However, there are 24 representations of a general point in that orthant. 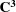 provides the possibility of choosing a particular region of the fundamental unit as the asymmetric unit where there is only a single representation of the general point (similar to an asymmetric unit in a space group).

The three components can be sorted by their magnitude. The second step is to exchange the real and imaginary parts of 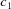 so that the real part is less than or equal to the imaginary part (if necessary); that requires also exchanging 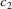 or 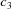. Finally, 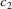 has its real and imaginary parts exchanged if necessary and of course those of 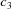 also. Note that the ordering of the real and imaginary parts of 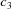 is not defined.


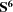
 does not provide a comparable simple suggestion for an asymmetric unit with a single unique representation of each lattice, except by converting to 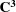 and back.

## 4. Measuring distance   

We require a distance metric that defines the shortest path among all the representations of two points (lattices). Common uses of a metric for lattices are searching in databases of unit-cell parameters, finding possible Bravais lattice types, locating possible suitable molecular replacement candidates and, recently, clustering of the images from serial crystallography.

A simple example of the complexity of the task is that we must decide which of the 24 reflections of one of the points is the closest to the other point. Using the reduction operations so that other paths are examined is also required. That the reduction operations do not commute means that the order of operations may in some cases be important.

It is also important to note that the necessary examination of reflections in calculating a distance may undo any time savings achieved by identification of unique cells in an asymmetric unit, so it is usually better to work in the full fundamental unit, rather than restricting our attention to the asymmetric unit. In the current work, the full fundamental unit is always considered.

The non-diagonal nature of reduction operations in this space means that measuring the distance between points in different regions of space is not as simple as finding the Cartesian distance. The edge-transform matrices transform a point in the fundamental unit to another, non-reduced unit, one where one scalar is positive. (Continued applications of the matrices will generate one or two more positive scalars.) Because of the non-diagonal nature of the matrices, the metric direction will change between each unit. The simple Euclidean distance from a point in the fundamental unit to one in another unit is not necessarily the minimal distance. A path broken by reflections and reduction transformations may be shorter. We present two alternative algorithms that do find a valid minimal distance. See Sections 4.1 and 4.2.

### 4.1. Measuring distance: virtual Cartesian points (VCPs)   

#### 4.1.1. Creating virtual Cartesian points   

**Figure 1:**
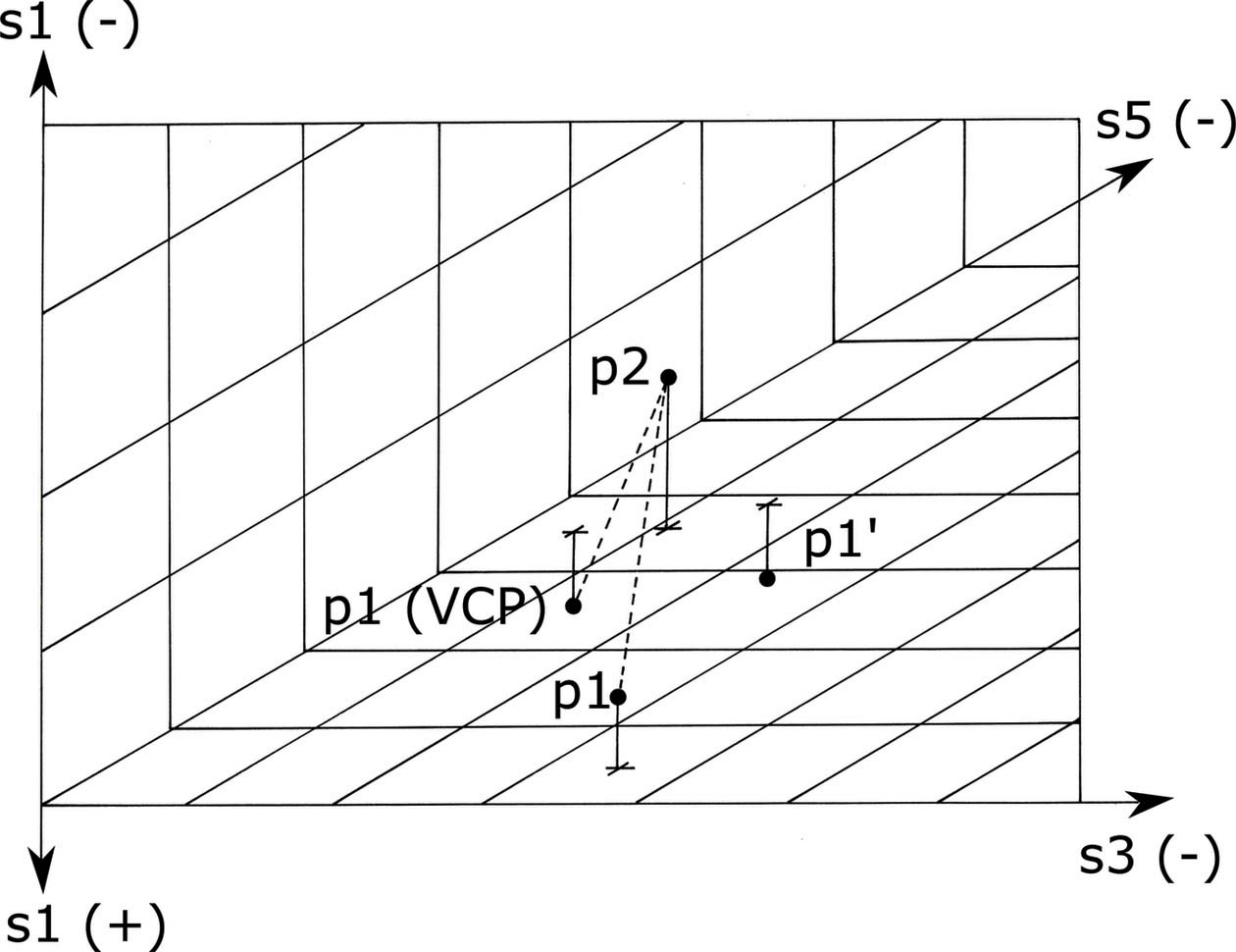
Example of a one-boundary virtual Cartesian point distance calculation. Only the VCP operation is shown, no reflections. Both points 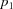 and 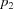 are Selling reduced. The image is the three-dimensional, all-negative octant of the three 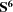 axes, 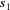, 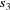 and 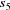; the reduction is done along the 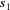 axis, and 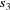 and 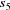 are the two scalars that will be interchanged. The points are shown above or below the 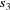/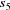 plane, with their projections onto that plane marked with a +. To compute the minimal distance between 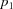 and 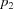, begin by computing the Euclidean distance between the two. The 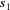 reduction transforms 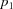 into 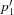, but the metric changes when going from negative 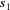 to positive 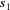, so the simple Euclidean distance may not be minimal. To generate 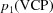 to which the distance may be shorter, project 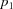 onto the 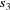/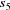 plane, transform that projected point, and subtract 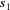 from that point. The distance from 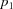 to 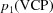 can now be used to decide whether it is shorter than the 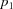 – 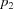 Euclidean distance. The best distance for this case is the shorter of the distances between 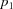 and 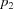 as opposed to the distance between 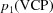 and 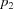.

For a point and a chosen operator for the reduction, we separate the point into two vectors: the projection onto the polytope for which the reduction axis is zero and the perp, the projection onto the reduction axis. The reduction operation is applied to the boundary-projected vector, and then the negative of the perp is added to that result. We call that resulting point the VCP (see Fig. 1 ▸). The goal of creating a VCP is that in measuring distances to points in the fundamental unit one can use the Euclidean metric of the fundamental unit.

#### 4.1.2. Using VCPs to determine distance   

**Figure 2:**
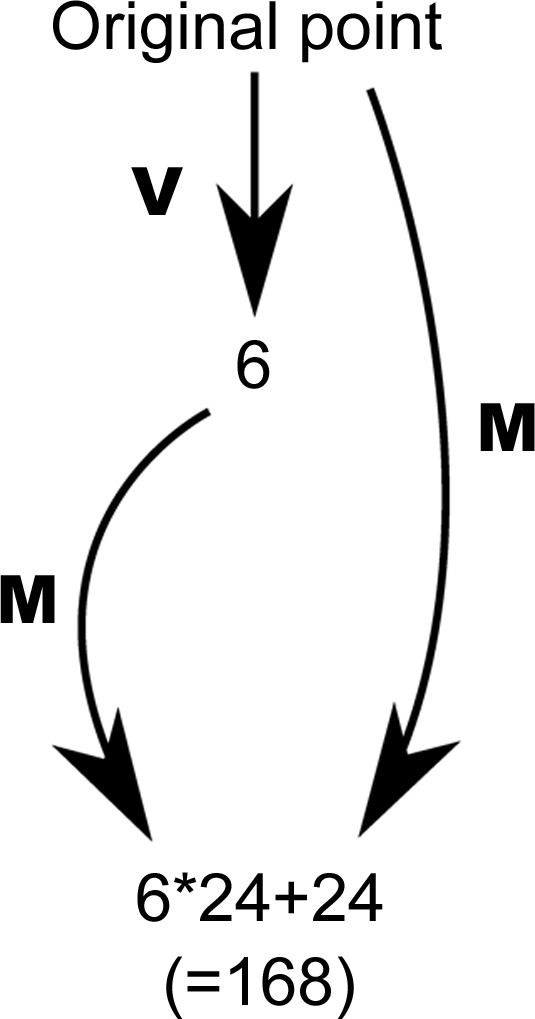
In this figure, **M** represents the application of all 24 reflection matrices. **V** represents the generation of six virtual Cartesian points from an input point.

To begin, the six VCPs (one for each boundary) are computed for the first of the two input points. Then the 24 reflections are computed for those six results plus the initial point itself. The desired distance is the minimum of the distances between the second point and all of the 168 points created in the first step. This is a one-boundary case. Monte Carlo experiments show that fewer than 1% of the minimal distances can be improved by two-boundary solutions and in most cases the difference is less than 10% (see Fig. 2 ▸).

Two-boundary solutions are created by first generating the same 168 points as in the-one boundary solution. Then we generate the six VCPs of the second input point and find the minimal distance between the 168 versus the first point and the seven points consisting of the six VCPs and the first point.

### 4.2. Measuring distance: tunneled mirrored boundaries   

**Figure 3:**
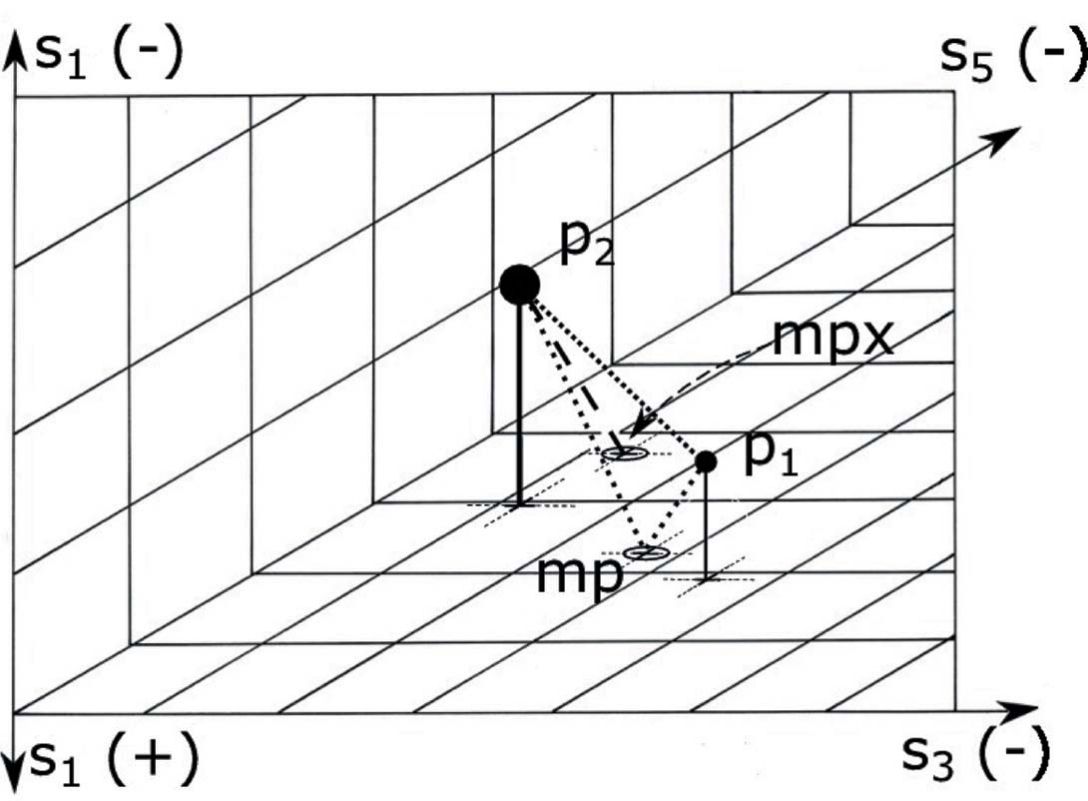
This an example of a one-boundary tunneled mirrored boundary distance calculation. As with Fig. 1 ▸ the 24 reflections are not shown. Both points 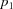 and 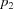 are Selling reduced. The image is the three-dimensional, all-negative octant of the three 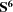 axes, 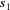, 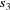, and 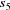; the reduction is done along the 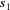 axis, and 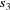 and 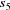 are the two scalars that will be interchanged. The points are shown above the 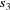/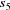 plane, with their projections onto that plane marked with a circled ‘X’. The Euclidean distance from 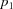 to 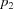 is shown as a dotted line. Let *mp* be the mirror point on the boundary going from 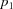 to 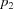 via the boundary. Then the shortest distance from 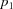 to *mp* to 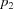 is also shown as a dotted line. The transformed image of *mp* is *mpx*. The distance between 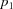 and *mp* is the same as the distance between a transformed 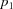 and *mpx*. There is a no-cost tunnel from *mp* to *mpx*. So the total alternative distance for this case is the distance between 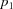 and *mp* plus the distance from *mpx* to 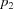 (shown as a dashed line).

An alternative to computing VCPs outside the fundamental unit is to compute mirror points in the boundaries and to tunnel between them with the boundary transformations. Start with points 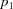 and 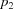 and one boundary *bd*, with projector 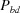; *e.g.* in Fig. 3 ▸ the *bd* is 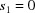. A simple mirror for a path from 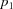 to *bd* and then to 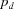 can be constructed from the hypo­tenuses of the two right triangles with heights equal to the distances from 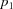 to *bd* and 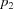 to *bd*, respectively, and legs made by dividing the line from 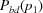 to 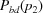 in the same proportions. Shorter paths may result by replacing the simple mirror point *mbd* with its image *mbd*′ under a boundary transformation and applying the 24 reflections both to the mirror point and to its transformation.

More general tunneling of this type is possible using two boundaries *bd_dwn_* with projector 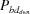, and 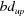 with projector 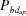


### 4.3. Measuring distance: example   

**Figure 4:**
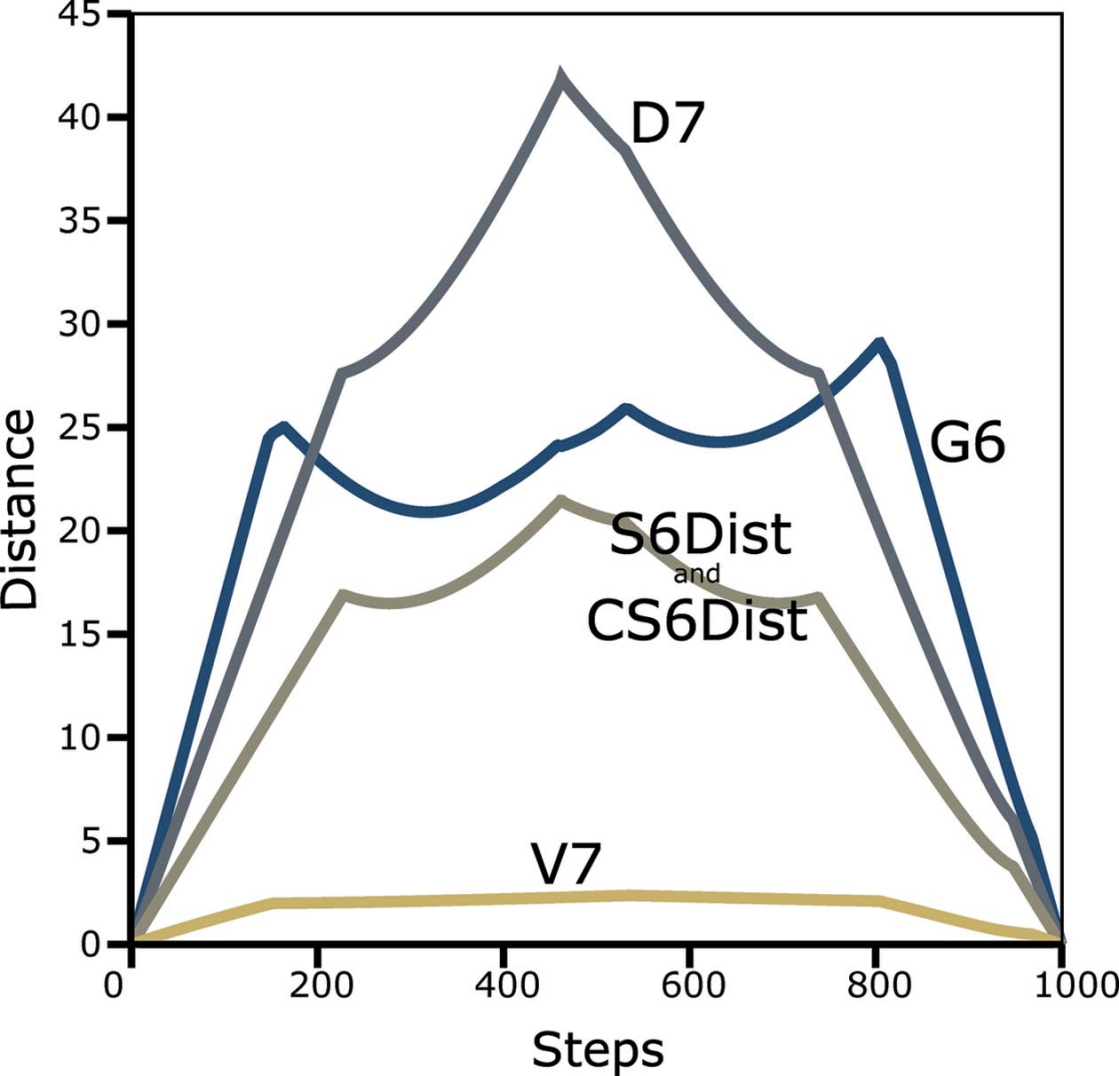
Distance between points using the *Follower* algorithm. To verify the distance algorithms, the ‘*Follower*’ algorithm has been developed. *Follower* chooses two points and determines the distance between one of them and all of the points on a line between the two original points. Here, one unreduced point is chosen and the second point is the reduced point of that point. So the distance between the original point and the final point is zero. Distances are shown for the 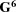 metric (Andrews & Bernstein, 2014 ▸), the 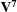 metric (Andrews *et al.*, 1980 ▸), the 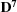 metric (Andrews *et al.*, 2019 ▸), and the two implementations in 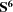. Timing in ms: 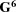 (NCDist) 4542, 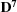 676, 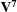 7, S6Dist 394, CS6Dist 14.

In order to verify the correctness and completeness of the implementations of distance algorithms, the ‘*Follower*’ algorithm was developed. It is implemented in the program *PointDistanceFollower*. Two points are chosen, a line constructed between them and then distances are calculated from each point along the line to the final point. One of the choices in the program is to make the final point be the reduced point of the starting point. The program also provides timing for the various options (see Fig. 4 ▸). Several criteria for quality control can be applied, such as: zero distance at both ends of the scan, continuity and only occasional discontinuities in slope (due to boundary crossings). This figure compares results from four metrics: 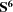, 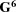, 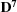 and 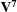.

It can be observed that the 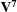 metric as seen in Fig. 4 ▸ is both fast to compute and smooth, and that leads one to ask whether 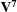 should not be the favored metric. The issue seems to have not been described well in the literature. For crystallographic purposes, a smooth metric is not sufficient. We also need sensitivity to the differences among lattices, especially for clustering.

The 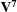 metric (Andrews *et al.*, 1980 ▸) was developed for the purpose of searching databases of unit-cell parameters. It was developed again by Rodgers & LePage (1992 ▸). The designation as 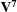 began in the work of Andrews & Bernstein (2014 ▸). The elements of the 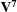 metric are: the reduced cell lengths, the reciprocals of the edge lengths of the reduced reciprocal cell, and the cube root of the volume of the primitive cell. Note that this definition means that each element has the same units. Because the edge lengths of reduced cells are stable to pertubation (Andrews *et al.*, 1980 ▸) and the primitive unit-cell volume is an invariant of the lattice (Andrews & Bernstein, 1995 ▸), we can be assured that the 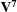 metric is stable to perturbation. In fact, this stability has led to systems where searches are only done using reduced cell edge lengths (and perhaps volume) (Mighell & Karen, 1996 ▸); obviously such searches have little to no sensitivity to angle differences.

The core problem with the 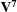 metric is that the sensitivity to angles decreases as angles approach 90°. This issue appears because of the definition of reciprocal cell parameters. For example, the reciprocal cell parameter a* is defined as: 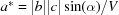, where *V* is the volume of the unit cell.

The issue that arises is that the sine function varies slowly in the neighborhood of 90°. Sensitivity to angle (cosine, the derivative of sine) approaches zero as the angle approaches 90°. Unfortunately for us, 90° angles are common in crystals, rendering 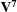 an insensitive metric for important regions.

Three issues can be seen immediately. First, if the derivatives are approaching zero, least-squares in 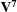 is likely to not perform well in some cases. Second, in the case of database searches, false-positive reports will be common. For example, Byram *et al.* (1996 ▸) explicitly describe the problem:

‘*Algorithms are designed to ensure that no known unit cells are missed in the search. The output may sometimes present numerous candidates for a match, but this can be screened readily by the researcher and is not considered problematic since the search is done only once per new crystal studied*’.

Third, in clustering, the failure of 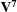 to distinguish lattices near 90° can prevent us from creating reasonably homogeneous clusters that can be distinguished with 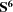, 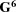 or 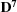. Of those three, 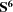 is the fastest.

## 5. Clustering   

This is a time of disruptive change in the image-clustering methods used in structural biology to understand polymorphs and dynamics at X-ray free-electron lasers and at synchrotrons. Serial crystallography is an essential technique at X-ray free-electron laser (XFEL) light sources and has become an important technique at synchrotrons as well (Rossmann, 2014 ▸), especially at newer high-brilliance beamlines. Methods that distribute the many diffraction images into clusters that likely represent crystals composed of proteins in similar states allow one to separate polymorphs and to categorize their dynamics. The inexorable increases in brilliance of these sources drives us to seek continual improvement in our algorithms and pipelines.

Clustering based on cell parameters is effective at the early stages of clustering when dealing with partial data sets. Here the Andrews–Bernstein NCDist cell-distance method (Andrews & Bernstein, 2014 ▸) used by Zeldin *et al.* (2015 ▸) is effective. One might investigate other criteria such as differences of Wilson plots to measure similarities of data (Foadi *et al.*, 2013 ▸). When the original data are complete (>75% today for similar applications), or one wants to achieve higher levels of completeness, one can cluster on correlation of intensities (CC, which stands for ‘correlation coefficient’) (Bernstein *et al.*, 2017 ▸). Changing the space being used from 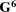 with NCDist to 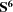 provides a significant performance improvement.

While NCDist has been effective for clustering, the original implementation is very demanding of computational resources. The development of CS6Dist, a macro-based 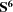 cell distance method, has improved cluster timing, both indirectly for NCDist by first reducing with 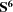 before finishing with Niggli reduction, and directly by computing 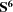 distances in which only six boundaries need to be considered instead of 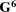 distances in which 15 boundaries need to be considered. Use of 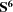 distances results in identical or qualitatively very similar dendrograms of cluster candidates obtained using 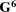. For example, the commonly used CCP4 clustering program *Blend* (Foadi *et al.*, 2013 ▸) has been modified to use 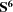 reduction and CS6Dist distances and tested on a set of 71 lysozyme 5° wedges from a slightly doped crystal, comparing NCDist and CS6Dist timing, on a 12-core, 24-thread AMD Ryzen thread­ripper system. The NCDist run took 28 s real time and 72 s user time. The CS6Dist run took 25 s real time and 40 s user time. The results were identical. This example and more challenging examples of the application of 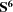 in clustering will be discussed in more detail in a subsequent paper.

## 6. Summary   

We have presented representations of a space (parameterized as 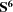 and 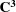) based on the Selling parameters and using the Selling reduction. Geometrically, this represents a significant simplification compared with the complex, non-convex asymmetric unit of Niggli reduction and 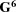.

Conceptually, there is simplification due to the orthogonal rather than inclined axes and single type of boundary of the reduced cell fundamental unit. Reasoning is simpler in such a Cartesian system. For one thing, there are fewer and simpler boundaries to the fundamental unit.

Distance calculations are faster in 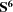 than in 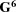. This is due to the simpler structure of the space which leads to simpler algorithms. Niggli reduction sorts the cell parameters, eliminating the 24-fold ambiguity that remains in Selling reduction. However, that advantage disappears when computing distances because it is still necessary to examine the same edge cases. Selling reduction saves time both for the reduction, and, more importantly, for the calculation of distances among lattices in lattice identification, in cell databases, and in cell clustering.

## 9. Availability of code   

The C++ code for distance calculations in 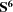 is available using https://github.com/; for CS6Dist.h, use https://github.com/yayahjb/ncdist; for *PointDistanceFollower* (*Follower* implementation), S6Dist.h and .cpp, use https://github.com/duck10/LatticeRepLib. 

## Figures and Tables

**Table 1:** The reflections in 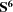 The 24 equivalent positions (Andrews *et al.*, 2019 ▸) in 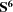 as matrices, given in the same order as the reflections in Table 2 ▸.

[	100000	/	010000	/	001000	/	000100	/	000010	/	000001	]
[	100000	/	001000	/	010000	/	000100	/	000001	/	000010	]
[	100000	/	000010	/	000001	/	000100	/	010000	/	001000	]
[	100000	/	000001	/	000010	/	000100	/	001000	/	010000	]
[	010000	/	100000	/	001000	/	000010	/	000100	/	000001	]
[	010000	/	001000	/	100000	/	000010	/	000001	/	000100	]
[	010000	/	000100	/	000001	/	000010	/	100000	/	001000	]
[	010000	/	000001	/	000100	/	000010	/	001000	/	100000	]
[	001000	/	100000	/	010000	/	000001	/	000100	/	000010	]
[	001000	/	010000	/	100000	/	000001	/	000010	/	000100	]
[	001000	/	000100	/	000010	/	000001	/	100000	/	010000	]
[	001000	/	000010	/	000100	/	000001	/	010000	/	100000	]
[	000100	/	010000	/	000001	/	100000	/	000010	/	001000	]
[	000100	/	001000	/	000010	/	100000	/	000001	/	010000	]
[	000100	/	000010	/	001000	/	100000	/	010000	/	000001	]
[	000100	/	000001	/	010000	/	100000	/	001000	/	000010	]
[	000010	/	100000	/	000001	/	010000	/	000100	/	001000	]
[	000010	/	001000	/	000100	/	010000	/	000001	/	100000	]
[	000010	/	000100	/	001000	/	010000	/	100000	/	000001	]
[	000010	/	000001	/	100000	/	010000	/	001000	/	000100	]
[	000001	/	100000	/	000010	/	001000	/	000100	/	010000	]
[	000001	/	010000	/	000100	/	001000	/	000010	/	100000	]
[	000001	/	000100	/	010000	/	001000	/	100000	/	000010	]
[	000001	/	000010	/	100000	/	001000	/	010000	/	000100	]

**Table 2:** The reflections in 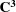 The 24 equivalent positions (Andrews *et al.*, 2019 ▸) in 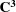 as permuations and real–imaginary exchanges (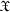), given in the same order as the equivalent matrices in Table 1 ▸.

[	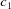 ,	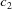 ,	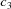	]	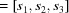	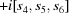
[	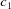 ,	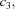	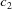	]	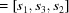	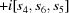
[	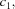	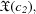	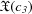	]	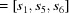	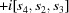
[	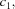	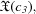	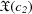	]	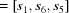	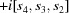
[	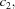	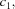	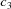	]	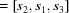	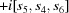
[	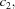	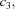	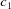	]	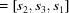	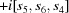
[	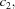	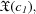	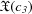	]	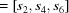	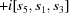
[	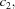	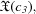	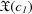	]	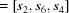	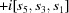
[	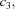	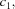	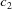	]	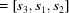	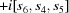
[	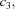	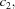	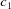	]	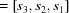	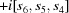
[	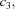	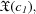	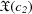	]	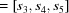	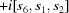
[	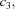	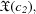	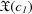	]	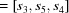	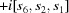
[	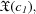	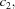	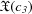	]	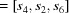	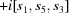
[	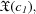	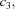	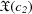	]	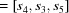	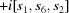
[	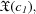	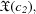	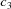	]	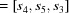	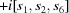
[	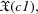	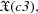	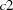	]	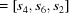	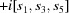
[	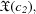	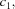	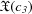	]	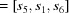	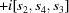
[	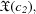	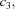	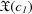	]	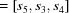	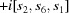
[	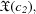	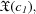	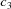	]	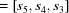	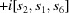
[	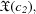	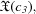	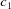	]	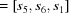	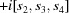
[	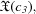	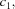	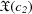	]	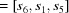	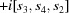
[	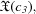	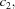	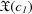	]	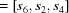	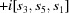
[	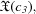	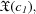	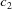	]	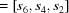	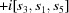
[	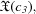	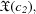	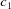	]	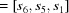	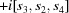
